# The development of non-destructive sampling methods of parchment skins for genetic species identification

**DOI:** 10.1371/journal.pone.0299524

**Published:** 2024-03-20

**Authors:** Melissa Scheible, Timothy L. Stinson, Matthew Breen, Benjamin J. Callahan, Rachael Thomas, Kelly A. Meiklejohn

**Affiliations:** 1 Department of Population Health and Pathobiology, College of Veterinary Medicine, North Carolina State University, Raleigh, North Carolina, United States of America; 2 Department of English, College of Humanities and Social Sciences, North Carolina State University, Raleigh, North Carolina, United States of America; 3 Department of Molecular Biomedical Sciences, College of Veterinary Medicine, North Carolina State University, Raleigh, North Carolina, United States of America; University of the Punjab, PAKISTAN

## Abstract

Parchment, the skins of animals prepared for use as writing surfaces, offers a valuable source of genetic information. Many have clearly defined provenance, allowing for the genetic findings to be evaluated in temporal and spatial context. While these documents can yield evidence of the animal sources, the DNA contained within these aged skins is often damaged and fragmented. Previously, genetic studies targeting parchment have used destructive sampling techniques and so the development and validation of non-destructive sampling methods would expand opportunities and facilitate testing of more precious documents, especially those with historical significance. Here we present genetic data obtained by non-destructive sampling of eight parchments spanning the 15th century to the modern day. We define a workflow for enriching the mitochondrial genome (mtGenome), generating next-generation sequencing reads to permit species identification, and providing interpretation guidance. Using sample replication, comparisons to destructively sampled controls, and by establishing authentication criteria, we were able to confidently assign full/near full mtGenome sequences to 56.3% of non-destructively sampled parchments, each with greater than 90% of the mtGenome reference covered. Six of eight parchments passed all four established thresholds with at least one non-destructive sample, highlighting promise for future studies.

## Introduction

Throughout the medieval period (c. 500–1500 AD), parchment served as the primary substrate for written texts across Europe and the Mediterranean world. The skins of animals (usually calf, sheep, or goat) were specially prepared for writing via a process of first dehairing by soaking in a bath containing lime and water, and then stretching on a wooden frame while scraping to the desired thinness using metal blades. More than one million books survive from the medieval period in addition to countless parchment documents such as correspondence and legal records. With the advent of printing (c. 1450 AD) most books were produced on paper, but the use of parchment was retained for civil and judicial documents well into the modern era due to the perception that it was more durable, and thus permanent [1]. While medieval books have traditionally been conceived of as repositories of textual and historical information, humanists, scientists, and librarians are also increasingly aware of the potential of their significant stores of biological information. This includes genetic traces left through both human and faunal contact. The study of the biological information stored in books is an emerging field known as "biocodicology".

Established methods for species identification of animal skins used in parchment have relied on various techniques, such as assessing the microscopic arrangement of hair follicles [2–5], determining the optical properties of the parchment [6], or performing protein analysis via peptide mass fingerprinting [7]. More recently, genetic methods have been incorporated into species identification studies, typically harnessing informative regions of the maternally-inherited mitochondrial genome (mtGenome), which has been well-characterized in many species [8–17]. In order to accommodate the degraded nature of parchment DNA, previous studies implementing DNA-based approaches for species identification of parchment have focused on amplifying regions from the mtGenome that are under 200 bp in length [18–20], with the resulting sequence(s) searched against a database of known species to determine origin. With the advent of newer ‘next-generation’ sequencing (NGS) technologies, complete mtGenomes have been generated from 2,000-year-old Dead Sea Scrolls to permit species identification [21]. While these previous studies showed some success in species identification, all focused on using cut pieces of parchment or destructive sampling of specimens for input into DNA analysis workflows. Neither of these approaches are amenable to testing precious and irreplaceable cultural heritage artifacts. One exception was Teasdale et al. [22], which utilized a non-invasive sampling technique previously described in Fiddyment et al. [7] to collect cells from a 1,000-year-old parchment for shotgun sequencing. While the sequence data generated in their study did successfully result in species identification for the 14 documents sampled, the authors reported very low endogenous DNA, and an average of only 19.3% (range = 0.7 to 51.4%) of generated sequences originated from the parchment source skin [22].

Emerging technologies incorporating NGS are expanding the possibility for the analysis of samples containing low-quality and low-quantity DNA. Hybridization capture (hyb capture), in which target biotinylated RNA baits are used to isolate complementary DNA fragments, is a promising technology for complex and challenging samples. Firstly, a single hyb capture assay is extremely versatile, as such assays can contain >200,000 short (~80 bp) baits, designed from species and locus specific reference sequences. This is particularly beneficial for parchment, since a single custom hyb capture panel can target full mtGenomes of multiple possible species commonly used for parchment production. Such a panel streamlines laboratory processing, allowing a single workflow to be used for any parchment. Secondly, baits are able to hybridize to template fragments with as little as 80% similarity, tolerating some flexibility for variation between historical animals used to furnish the skins and the modern references used to design the baits. Hyb capture has even shown success in recovering the mtGenome of a closely-related species (*i*.*e*., same genus) to the designed baits (e.g., [23–26]). Thirdly, many short baits that bind across the entire length of a locus can be included, meaning that unlike traditional PCR, data recovery is not reliant on full length DNA templates. Lastly, while PCR-based methods target a single short region at a time, simultaneous enrichment of the full mtGenome using the hyb capture assay may eventually allow for geographic origin estimates of the animals sourced for the parchment, if a thorough database of known mtGenomes throughout time and location can be established. Hyb capture combined with NGS has permitted the recovery of full mtGenomes from challenging samples, particularly those with highly fragmented DNA (average length ~50–70 bp) [27–29] and those exposed to prior chemical treatment [28, 30]. Additionally, hyb capture was performed on three of the 2,000-year-old Dead Sea Scrolls fragments mentioned above, using baits from mtGenomes of the target parchment species (cow, goat, and sheep), which confirmed the results generated with the non-targeted sequencing [21].

Parchment documents can be an abundant source of genetic information, permitting the characterization of animals throughout history accompanied with detailed records of time and location. Notably however, the very nature of parchment means that DNA analysis is not straight-forward for several key reasons: 1) the chemicals used during the parchment making process [31] and the age of these documents [22, 32–34] contribute to the degraded nature of the endogenous DNA, 2) mixtures of multiple species can be present, whether through human touch, surface treatments, or sample to sample contamination during parchment-making process itself (e.g., multiple skins soaked in the same lime bath) [31, 31, 35], and 3) the individuals used to make the skins are from historical breeds, such that appropriate mtGenome reference sequences are not readily available. To unlock the information contained within parchment documents, this study documents a) the testing and development of non-destructive sampling techniques to collect cellular material from parchment skins, and b) a wet-laboratory protocol harnessing hyb capture and NGS to generate full mtGenome sequences for species identification.

## Materials and methods

**Fig 1:**
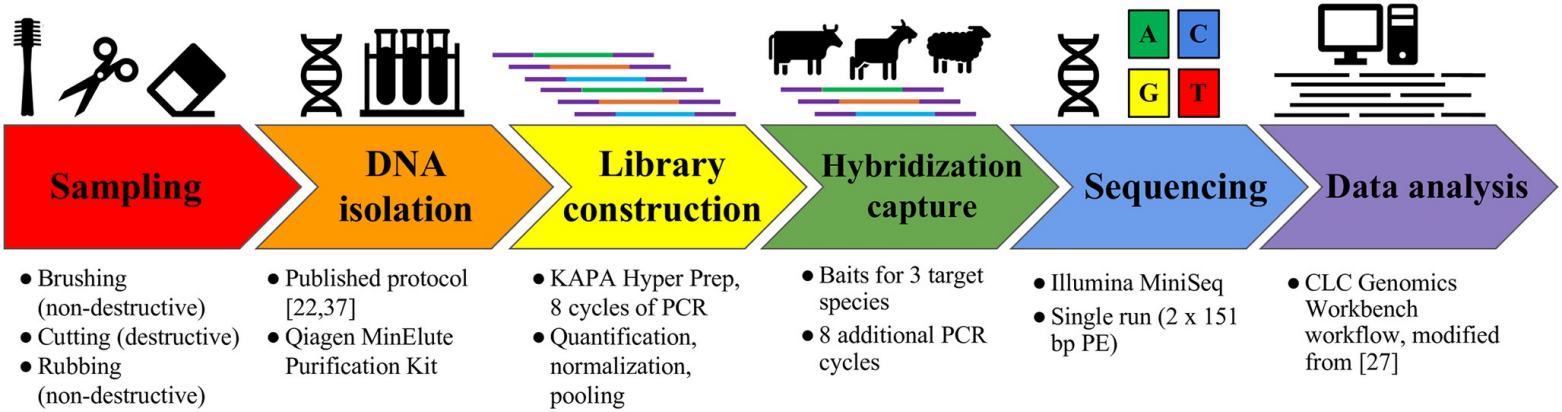
Experimental design for collection and processing of parchment samples for genetic analysis. After sampling (both non-destructive and destructive), DNA was isolated. NGS libraries were constructed from all available genomic DNA and subsequently enriched for mitochondrial DNA via hybridization capture using commercial baits designed from the cow, goat, and sheep mtGenome sequences (Daicel Arbor Biosciences). The enriched libraries were sequenced on an Illumina MiniSeq (Illumina), and resulting reads were processed in a custom workflow within the CLC Genomic Workbench (Qiagen). Four criteria were implemented to authenticate the source species of the parchment.

A general schematic of the experimental design of this project is given in Fig 1. Details for each step are given below.

### Parchment selection and sampling

**Fig 2:**
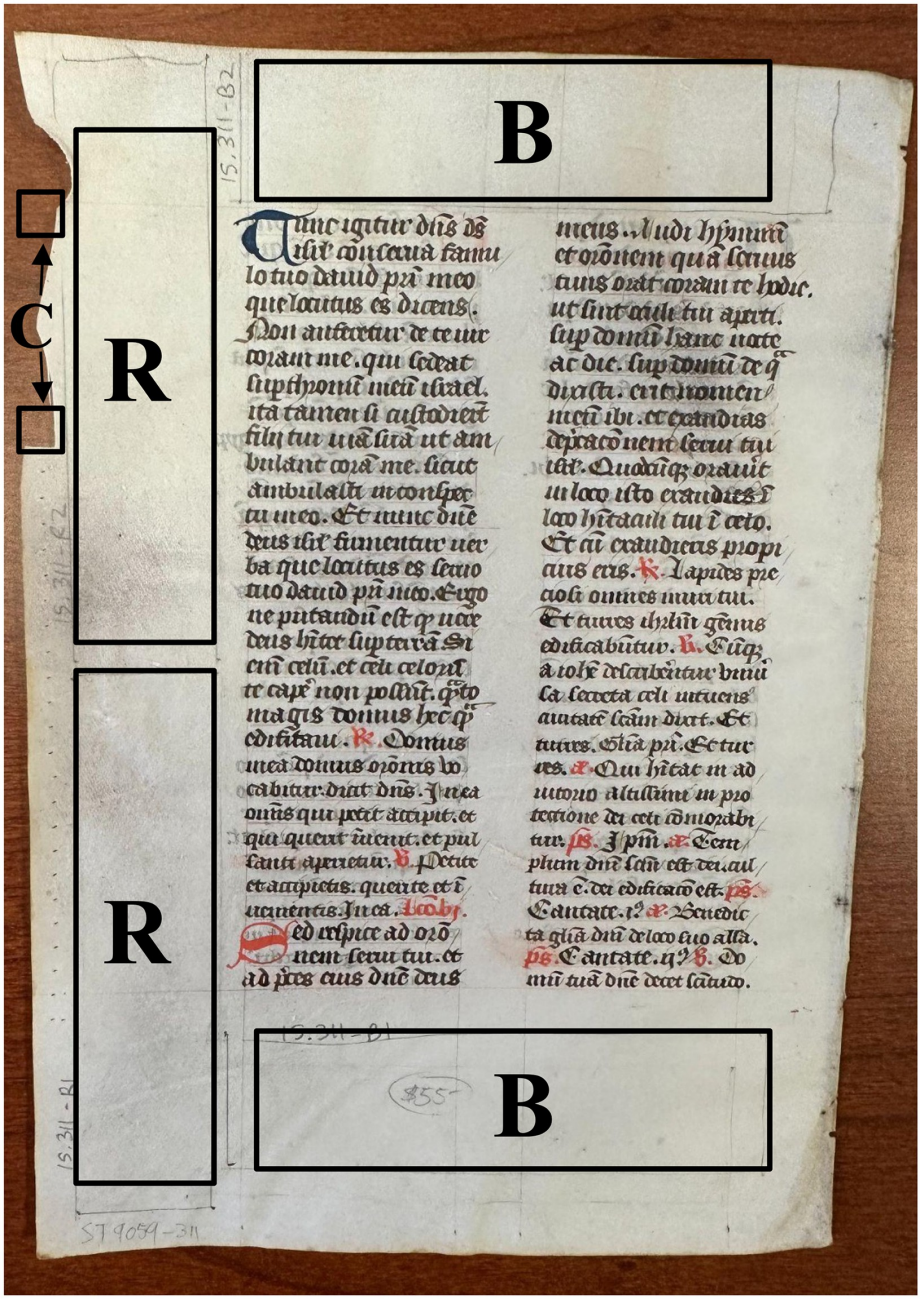
Strategy for sampling both destructively (cutting, “C”) and non-destructively (brushing, “B”; rubbing, “R”) from parchment documents. Duplicate samples of each method were collected from each document. The parchment shown is 15–311.

A total of eight documents (Table 1) made of parchment were obtained for sampling, seven ranging in age from the 15^th^ to the late 19^th^ century (herein referred to as “historical”) and an additional “modern” parchment made in 2012 from a known source (cow, *Bos taurus*) that acted as a positive control. No permits were required for the described study, which complied with all relevant regulations. Two of the historical parchments (leaves taken from books), were already owned (T. Stinson’s personal collection), as was the modern parchment. The remaining five historical parchments were purchased from a reputable dealer and were legal documents (dated and localized) consisting of a large piece of folded parchment (Table 1). To limit variability, each document was visually inspected under a microscope to identify the “hair side” (*i*.*e*., the side of the skin that previously had hair), and the opposite “flesh side” was targeted for sampling. Four sections (approximately 2.5 cm by 10 cm each) without text were marked with pencil on each document for non-destructive sampling (detailed below; Fig 2). When sampling the book leaves, areas avoided included those previously near the gutter (identified by stitching which may contain accumulation of debris), high-touch areas such as the outer bottom corners (likely containing human touch DNA) and areas of repair that may have incorporated non-original materials.

**Table 1:** Parchments sampled for genetic testing.

Parchment ID	Date of copying	Text	Place copied
*15–311*	1472	Excerpt from I Kings, Chapter 8	Central France
*1738*	1738	Indenture/mortgage	Suffolk, England
*1763*	1763	Legal document; booklet format	France
*1785*	1785	Indenture/release of dwelling	Devon, England
*1812*	1812	Indenture/lease	Norwich, England
*1840*	1840	Indenture/mortgage	Suffolk, England
*1894*	1894	Indenture/mortgage	Suffolk, England
*Modern*	2012	N/A	N/A (manufactured in Montgomery, NY)

Each document was sampled in duplicate using three different methods: 1) *Rubbing (non-destructive)*. Following a previously published technique [7, 22], Mars Plastic Erasers (Staedtler, Nuremberg, Germany) were purchased to complete sample collection by eraser rubbing, a technique commonly used by conservators to clean parchment. Each eraser was unpackaged immediately before collection and broken in half by a gloved hand. Using a single half of the eraser, the identified parchment surface (2.5 cm by 10 cm area) was gently rubbed to produce crumbs. A clean single-use disposable spatula (VWR International, Radnor, PA) was used to collect eraser crumbs and place them in a clean, labeled 1.7 mL DNA LoBind tube (Eppendorf, Hamburg, Germany). Sampling via rubbing continued in this manner until the crumbs filled up to the 0.25 mL mark of the tube. 2) *Brushing (non-destructive)*. In a technique previously identified [36], EndoCervex-Brushes (Rovers, Lekstraat, Netherlands) were used to collect cellular material from the surface of the parchment by gently brushing the bristles over a separate area (2.5 cm by 10 cm area) for approximately one minute, turning the brush as necessary to use all available bristles. Once collection was complete, the detachable brush head was placed directly inside a clean, labeled 1.7 mL LoBind tube. 3) *Cutting (destructive)*. A scalpel (Fisher Scientific, Hampton, NH) was used to cut an ~0.5 cm^2^ piece of parchment from the edge of each document (avoiding high touch areas). Each piece of cut parchment was placed directly into a clean 1.7 mL LoBind tube. The scalpel was rinsed with 10% bleach then 70% ethyl alcohol and allowed to dry between each sample. Notably, while destructive analysis is not recommended for future sampling, these cuttings acted as positive controls for each of the parchment documents.

Sampling was completed in a clean PCR hood, and all six samples (rubbing, brushing and cutting, each in duplicate) from a single parchment were collected one after another. Nitrile gloves were worn during sample collection. Before sampling from a new parchment, the PCR hood was disinfected using 10% bleach and exposed to ultraviolet light for 15 minutes, and the analyst changed gloves. In order to monitor possible contamination from the collection materials and sampling process, three separate blanks were collected on each day of sampling: 1) a clean fragment of eraser was rubbed against the inside of the eraser’s cardboard packaging to generate eraser crumbs, and those were placed inside a clean, labeled 1.7 mL LoBind tube, 2) a clean EndoCervex-Brush was directly placed into a clean, labeled 1.7 mL LoBind tube, and 3) solely a clean, labeled 1.7 mL LoBind tube. Samples and controls collected using all three methods were stored at room temperature prior to DNA isolation.

### DNA isolation

DNA isolation was performed in three batches of 24 or fewer samples using a published method [22, 37]. Briefly, an isolation buffer was prepared inside a clean PCR hood using defined ratios of ethylenediaminetetraacetic acid (0.05M EDTA; ThermoFisher Scientific, Waltham, MA), sodium dodecyl sulfate (0.08% SDS; Promega, Madison, WI), Proteinase K (0.5 mg/mL; Qiagen, Hilden, Germany), and DNase/RNase-free distilled water (ThermoFisher Scientific) reported in Teasdale et al. [22]. A total of 500 μL of the prepared buffer was added to each sample tube (containing either the eraser rubbing, brush or cutting) or blank tube, and incubated in a ThermoMixer (Eppendorf) for approximately 24 hours at 50°C and 800 rpm rotation. Once samples were removed from incubation, the remaining DNA isolation steps took place inside a dedicated biosafety cabinet to minimize contamination. Each sample was spun in a centrifuge (5 minutes at 13,000 rpm; 15,871 x g), the supernatant mixed with five volumes of Buffer PB (Qiagen), and the mixture carefully transferred to a MinElute Spin Column (Qiagen), in volumes up to 750 μL. Purification of DNA using the MinElute was used as described in the microcentrifuge protocol within the kit handbook [38]. DNA was eluted in 60 μL of pre-heated (65°C) Buffer EB with a 5-minute incubation before the elution spin. For every batch of DNA isolations, three additional isolation reagent blanks were included to assess possible contamination. When not in use, isolated DNA was stored at -20°C. The Qubit dsDNA High Sensitivity (HS) Assay Kit (ThermoFisher Scientific) on the Qubit Fluorometer 3.0 (ThermoFisher Scientific) was used to determine the quantity of total DNA isolated from each sample.

### Library construction

Libraries were constructed using the KAPA Hyper Prep Kit (Roche, Basel, Switzerland) using 50 μL of isolated DNA from samples or reagent blanks. An additional library negative was included with 50 μL of Buffer EB used as input. Custom dual-index adapters manufactured by Integrated DNA Technologies (Coralville, IA) and equivalent to TruSeq DNA CD Indexes (Illumina, San Diego, CA) were used to individually barcode each sample for sequencing. Eight cycles of amplification were performed within the library construction protocol, and all bead purifications were performed (at bead ratios recommended within the library construction protocol) using AMPure XP Beads (Beckman Coulter, Indianapolis, IN). Libraries were quantified using the KAPA Library Quantification Kit (Roche) and the QuantStudio 5 Real-Time PCR System (ThermoFisher Scientific). Measured library quantities were used to generate a single pool of all 58 libraries (48 samples, nine reagent blanks, and one library negative) with equimolar representation. For any sample or control that did not have a sufficient quantity, a maximum volume of 15 μL was added to the pool. Once libraries were pooled, a standard 1.8X bead purification with AMPure XP Beads (Beckman Coulter) was used to concentrate 200 μL of the pool into a smaller volume of 30 μL. The Qubit dsDNA HS Assay Kit (ThermoFisher Scientific) was used to determine the pre- and post-purification pool concentrations.

### Mitochondrial genome (mtGenome) enrichment and sequencing

The single pooled library was enriched with the myBaits Expert Mito v4 kit (Daicel Arbor Biosciences, Ann Arbor, MI) using a custom mix of three commercially available panels (cow, *Bos taurus*; domestic goat, *Capra hircus*; and domestic sheep, *Ovis aries*) following v4.01 of the user manual. A single round of hybridization was performed for the single pool at 60°C for ~43 hours. Eight cycles of PCR were completed post-hybridization, and the concentration of the enriched, amplified pooled library was measured using the Kapa Library Quantification Kit (Roche). The final library was sequenced on the MiniSeq System (Illumina) using the MiniSeq Mid Output Kit (Illumina), paired end sequencing (2 x 151 bp), a 25% PhiX spike (within the 10–50% recommended for the 2-channel chemistry instrument), and a 1.4 pM library loading concentration.

### Creating consensus mitochondrial genomes (mtGenomes)

Raw reads were imported into the CLC Genomics Workbench (Qiagen) and processed through a workflow based on the one described in Molto et al. [27]. Briefly, this workflow incorporated several key steps including a) merging overlapping read pairs and only carrying merged reads forward, b) read quality trim, using a quality score limit of 0.05, c) mapping of high-quality reads to a list of reference mtGenomes, d) removal of duplicate reads introduced during PCR (*i*.*e*., reads with identical start/stop positions and length), and e) local realignment. Notably in a modification to the workflow described by Molto et al. [27], in this study we included a total of sixteen references for the mapping (S1 Table): the three species represented in the myBaits panel (cow, goat, sheep; specifically, the exact references used to design the baits), a human reference to detect any human contamination, and several other mammals to detect reads of non-target species (*i*.*e*., those less commonly used in parchment, traditional pets that could be a contaminant source, and species that may be present in a glue or gelatin [35] and applied to the parchment surface). While non-mammalian (bacterial, fungal, *etc*.) contaminant reads are likely, the analysis of these reads was outside the scope of this study. Reads mapping equally to multiple references were ignored rather than mapped randomly. Reference mappings were sorted by number of reads aligned, and the details were recorded for each top non-human reference mapping. A multiple sequence alignment of references and samples, along with pairwise comparisons, were performed in the CLC Genomics Workbench. Statistical analyses (Student’s t and One-way ANOVA) and summary charts were generated in JMP Pro 16 and (SAS Institute, Cary, NC) and Excel v16.79 (Microsoft, Redmond, WA).

### Authentication of the sample mitochondrial genome (mtGenome) consensus sequence and species assignment

Four criteria were implemented to authenticate the consensus mtGenome sequence and species assignment for each sample as follows: 1) *Mean coverage mapping*. The total number of mapped reads across the entire mtGenome (~16,000 base pairs) was assessed. Read mapping and resulting consensus sequences yielded greater confidence if the coverage averaged greater than 10X across the entire reference. 2) *Percentage of mtGenome covered*. The percentage of the mtGenome covered by the consensus sequence was determined. Greater confidence in species assignment was placed on consensus sequences with greater than 90% of the reference recovered. Uneven coverage with large gapped regions would be more likely for reads mapping to the incorrect species. 3) *GenBank top match*. The mtGenome consensus sequence for each sample was searched using the default settings of a nucleotide BLAST search of the National Library of Medicine’s National Center for Biotechnology Information (NCBI) database. The best match based on E-value was determined to assign a source species, and the E-value was required to be <0.001. Percent identity >99% and query coverage > 95% also had to be achieved to pass this criteria. 4) *Phylogenetic tree placement*. The mtGenome consensus sequences were aligned with the sixteen reference mtGenomes (S1 Table) using the CLC Genomics Workbench (Qiagen). A phylogenetic tree was created in the CLC Genomics Workbench using the UPGMA algorithm and Kimura 80 distance measure with 1,000 bootstrap replicates to confirm placement within the tree. The generated tree was exported as Newick/Nexus files and imported into Dendroscope 3 for viewing and editing [39]. Any inconsistencies (*e*.*g*., a sample with a tentative identification as cow not clustering with the cow reference mtGenome in the tree) would flag a sample for further investigation. If a sample did not pass any of the four authentication criteria, it was not evaluated for subsequent criteria.

## Results and discussion

### DNA, library and sequencing yields

A total of 48 samples were collected across the eight parchments for downstream processing. Six samples had insufficient genomic DNA to permit quantification with the Qubit dsDNA HS Assay (lower limit of the assay is 0.005 ng/μL). The remaining 42 samples ranged in genomic DNA concentration from 0.053 to 30.8 ng/μL, with an average total yield of 202 ± 392 ng. The amount of genomic DNA used as input into library construction averaged 168.2 ± 326.6 ng, with a maximum input of 1,540 ng. The resulting individual library concentrations ranged from 5.4 to 393.1 nM (Table 2), with an average of 101.6 ± 101.1 nM. Significantly higher library yields were observed from the cutting than from either rubbing (p = 0.0089) and brushing (p = 0.0042), but there was no significant difference between two non-destructive methods (brushing and rubbing; p = 0.7763). After pooling all samples and controls (total n = 58) the concentration of the pooled library was measured at 4.79 ng/μL. Following the 1.8X AMPure XP cleanup to concentrate the library, the resulting concentration was 34.4 ng/μL for a total input into hybridization capture of 240.8 ng. After the hybridization capture enrichment, the final enriched library was determined to be 0.2 nM.

**Table 2:** Average summary metrics from the wet-lab processing for each sampling method (cutting, rubbing and brushing; collected each in duplicate) for the eight parchment documents sampled in this study. Abbreviations are as follows: nM, nanomolar; bp, base pair.

Parchment ID	Library concentration (nM)			Mapped reads (#)			Mean coverage/read depth			Reference covered (%)			Mean read length (bp)			Source species
	Brushing	Cutting	Rubbing	Brushing	Cutting	Rubbing	Brushing	Cutting	Rubbing	Brushing	Cutting	Rubbing	Brushing	Cutting	Rubbing	
*15–311*	18.0	165.0	26.7	246	11,234	1,225	1.9	65.2	8.2	80.7	100.0	98.7	125.3	94.7	111.7	*Bos taurus*
*1738*	69.7	196.7	49.6	7,704	28,581	12,818	59.8	184.8	90.0	100.0	100.0	100.0	129.9	108.3	116.8	*Ovis aries*
*1763*	30.3	152.5	82.0	629	17,219	1,551	4.6	112.5	8.6	94.4	100.0	98.7	119.8	109.4	93.0	*Ovis aries*
*1785*	59.6	142.0	71.7	6,183	38,763	5,447	43.1	273.9	35.8	99.8	100.0	99.7	116.6	118.6	110.2	*Ovis aries*
*1812*	42.6	190.6	23.1	394	5,466	812	2.8	34.9	5.2	91.2	99.8	97.2	118.4	105.7	107.2	*Ovis aries*
*1840*	7.0	316.0	11.5	5,154	40,353	13,036	41.0	350.6	107.3	100.0	100.0	100.0	132.4	145.7	134.1	*Ovis aries*
*1894*	9.0	77.8	8.9	1,758	27,773	1,370	9.7	158.7	7.4	96.4	99.8	97.6	92.7	95.2	89.4	*Ovis aries*
*Modern*	288.2	74.2	325.9	22,348	27,812	31,194	228.5	261.0	279.6	100.0	100.0	100.0	171.0	156.5	148.2	*Bos taurus*

The single cartridge yielded a total of 3,078,034 sample-assigned reads, while 49% of the total reads were generated from the PhiX spike-in. PhiX was likely overrepresented due to the extremely low final pooled enriched library concentration, despite the dilution adjustments made during preparation for sequencing. The number of raw reads generated ranged from 4,046 to 423,562 per sample, with an average of 64,126 ± 82,870 reads. Following the established data analysis workflow, duplicates were removed and ranged from 14–83% per sample, with an average of 34 ± 19%. The total of mapped reads to the top non-human reference ranged from 239 to 40,961 per sample, with an average of 12,878 ± 13,081 reads (Table 2). The destructive sampling generated significantly more mapped reads than either non-destructive method (brushing p<0.0001; rubbing p<0.0001). There was no significant difference in mapped reads between the non-destructive methods (p = 0.4272). Overall, gaps across mappings were minimal, and they were typically present in samples with low overall coverage and most commonly occurred over the highly variable control region (also known as the D-Loop). A statistically significant difference in percentage of the reference covered was observed both between the cutting and brushing methods (p = 0.0025), and between the non-destructive methods (brushing and rubbing; p = 0.0145), with cuttings providing the highest percentage and brushing providing the lowest percentage of the sampling methods. Only three samples (6.3%) did not have >90% of the mtGenome reference covered in the resulting consensus sequence. Notably, these three samples were collected only using the brushing method and were from the older historical parchment documents (15–311 and 1763; S2 Table). The mean read length of the mapped reads ranged from 86 to 175 bp, with an average of 119 ± 21 bp. No significant differences in mean read length were detected by sampling method.

The controls used to assess contamination (human or across-sample) during the wet-laboratory steps in this study were deemed as “clean”. The extraction reagent blanks containing a clean brush (n = 3) yielded only 48–167 mapped reads of which 81% of those reads mapped to the human mtGenome reference. Notably, the eraser rubbing extraction blanks (n = 3) were much cleaner, yielding only 2–10 mapped reads, of which 7% of those reads mapped to the human mtGenome reference. The reagent blanks consisting of only an empty tube and extraction reagents (n = 3), generated only 0–7 mapped reads, none mapping to the human mtGenome reference. Further, the library negative, introduced during library construction, contained zero mapped reads.

### Authentication criteria

**Fig 3:**
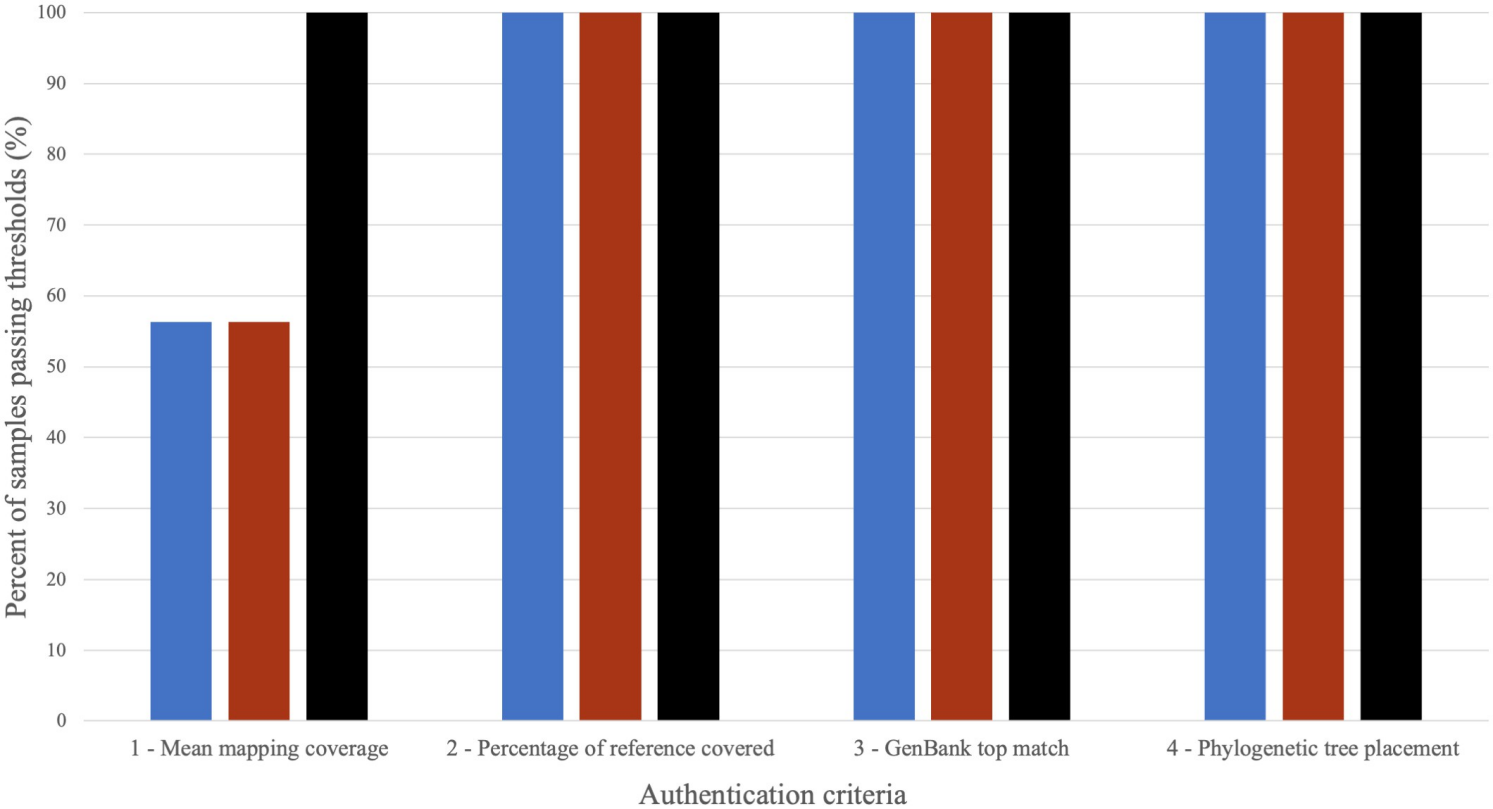
Summary of the performance of the different sampling methods for each of the four authentication criteria. Data from eight parchment documents collected in duplicate with each sampling method (total n = 48) were used to generate these summary statistics. If a sample failed one criterion, it was not evaluated for subsequent criteria. Rubbings are shown in blue, brushings are in red, and cuttings are in black.

Authentication of mtGenome consensus sequences and species assignment was performed using four criteria. Fig 3 shows the summary results for each authentication criteria for all the samples processed (total n = 48); breakdown per sample and parchment source is given in S2 Table.

**Fig 4:**
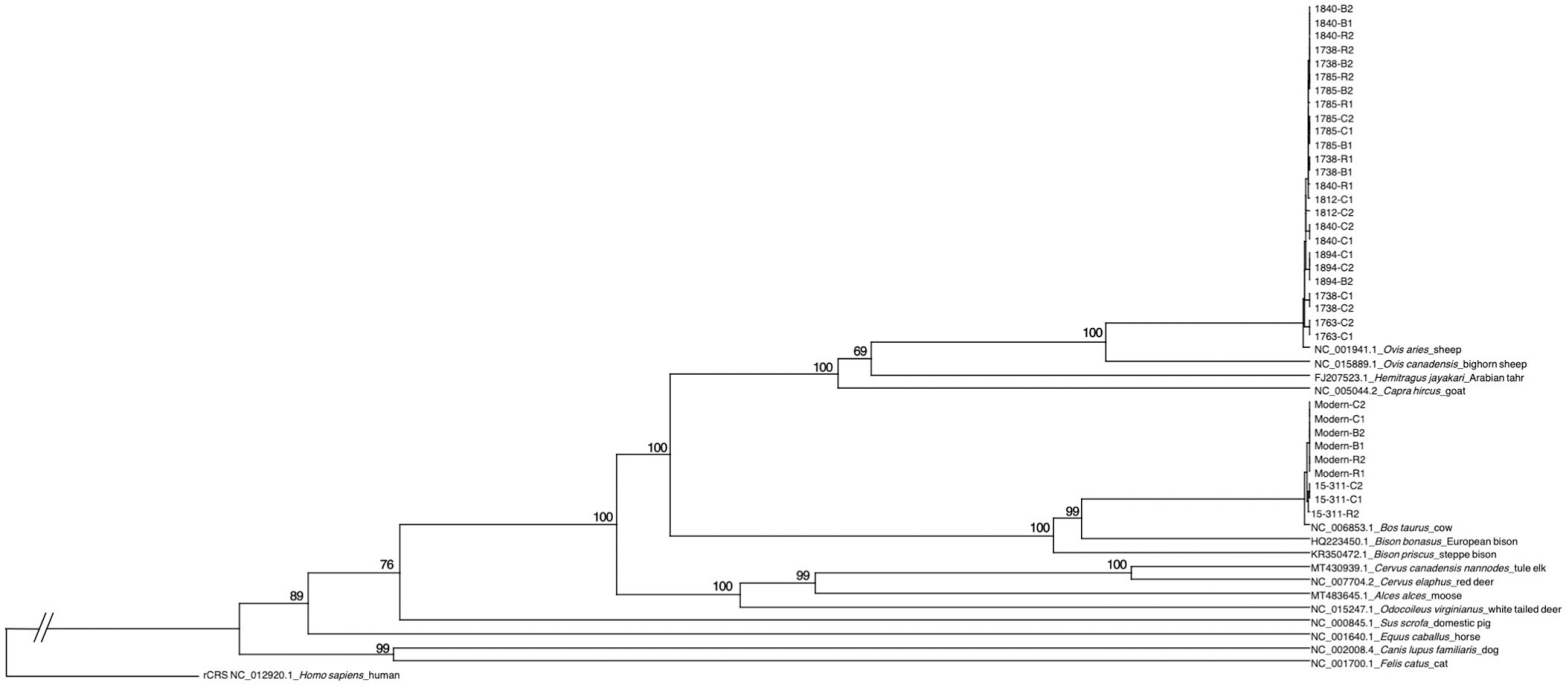
Phylogenetic tree showing the placement of the consensus mitochondrial genome (mtGenome) sequences generated from parchment documents in this study. The tree was reconstructed using the UPGMA algorithm and Kimura 80 distance measure with 1,000 bootstrap replicates. The human (*Homo sapiens*) reference serves as the outgroup, and bootstrap values are listed. References are named with the GenBank Accession number, followed by the scientific name, then then common name. Samples from the present study are named with the Parchment ID, collection method (C = cutting, R = rubbing, B = brushing) and replicate number.

1. *Mean mapping coverage*. A total of 14 samples (29%) fell below the 10 X mean mapping coverage threshold, and these were evenly split between samples collected using both non-destructive sampling methods (rubbing and brushing; S2 Table). After excluding these samples, the average (± standard deviation) mapping coverage for cutting (n = 14), rubbings and brushings (each n = 9) were 180 ± 106, 115 ± 103, and 85 ± 83, respectively. While there was a significant difference between cutting and brushing (p = 0.0288), there was not a difference between cutting and rubbing (p = 0.1295) and rubbing and brushing (p = 0.5203). Samples not passing this criterion were excluded from subsequent authentication.
2. *Percentage of reference covered*. For the samples evaluated for authentication (n = 34), the average percentage of the reference covered was broadly consistent for brushings (99.8 ± 0.3%), cuttings (99.9 ± 0.1%) and rubbings (99.9 ± 0.3%). There was no significant difference across methods for the percentage of reference covered (p = 0.3241 to 0.9540).
3. *GenBank top match*. In all evaluated consensus sequences (n = 34), the highest-ranking non-human reference genome mapping was consistent with the highest-ranking species match in GenBank. All consensus sequences returned a top match in GenBank with an E-value of 0, a query coverage of 100%, and the percent identity ranged from 99.8 to 100%.
4. *Phylogenetic tree placement*. Consensus sequences passing the previous authentication criteria (n = 34) were included in the phylogenetic tree analysis (Fig 4). We observed that in all evaluated samples, the mtGenome consensus sequences generated from parchment samples clustered in the tree with a) the top match species from the list of references (S1 Table) with the best mapping results, and b) the GenBank top match. Given branch lengths on a phylogenetic tree are reflective of the genetic divergence (or the number of mismatching nucleotides) between sequences, we examined whether samples from a single parchment collected using both destructive and non-destructive methods clustered together on the tree connected by short branch lengths. In general, replicates from each parchment clustered together, particularly with duplicates of the same sampling method. Both parchments determined to be from cow have all authenticated replicates clustered together (Fig 4). While some parchments identified as sheep do not have all replicates clustered together perfectly, the majority of the sheep mtGenomes represented in this study are >99.9% identical (S3 Table), and minimal coverage gaps may be the source of replicate separation within the tree.

### Species assignment

After evaluating the consensus mtGenomes from each sample against the four authentication criteria (available in S1 Appendix), we determined that six of the eight parchments tested in this study (1738, 1763, 1785, 1812, 1840, and 1894) are consistent with an *Ovis aries* (sheep) origin, while one (15–311) is consistent with a *Bos taurus* (cow/cattle) origin (Table 2). Our results confirmed the known truth origin of the Modern parchment as *Bos taurus* (cow/cattle) (Table 2).

### Detection of contaminants and non-target DNA

**Fig 5:**
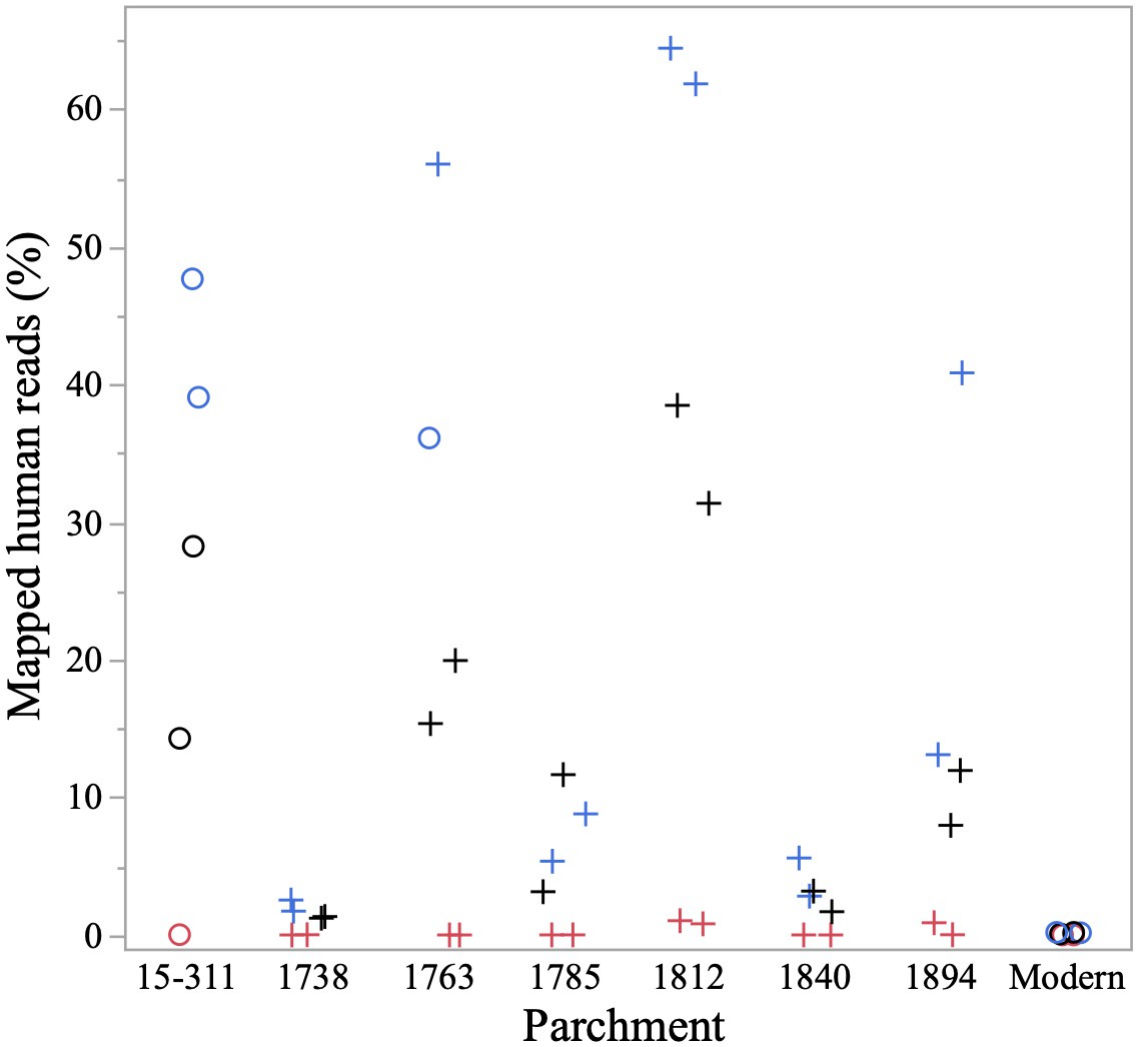
Percentage of total reads per sample that mapped to the human mitochondrial genome (mtGenome) reference. Parchment documents are arranged along the x-axis based on Parchment ID. Colors represent sampling method as either cutting (destructive, red), rubbing (non-destructive, black) and brushing (non-destructive, blue). Symbol shape denotes the non-human species with the highest number of reads mapping as either *Bos taurus* (cow/cattle, circle) or *Ovis aries* (sheep, cross). All 48 processed samples are represented with no exclusions due to authentication criteria.

While the baits used for hybridization capture were designed from the three target source species (cow, goat and sheep), DNA fragments with greater than ~80% identity with the baits can also be enriched. Therefore, given the conserved nature of the mtGenome across mammals [40, 41], DNA from non-target species can be recovered whether authentic to the parchment source or derived from surface contamination. We observed this most substantially with human contamination, likely due to touch DNA from handling of the parchments. This is most pronounced in the brushing samples with 24.1 ± 24.3% of reads mapping to the human mtGenome, with a smaller percentage of human reads represented in the rubbings (11.9 ± 12.1%), a significant difference (p = 0.0323). Notably, contaminant human reads are nearly absent in cuttings (0.2 ± 0.4%) (Fig 5), significantly lower than brushings (p<0.0001) and rubbings (p = 0.0407). Additionally, the level of human contamination appears to be parchment-specific; parchments 1738, 1785, 1840, and the modern parchment (Fig 5) each had less than 15% of assigned reads mapping to the human mtGenome reference, whereas parchment 1812 had >60% assigned reads from brushing samples mapping to the human mtGenome reference.

### Mean read length

While we did not include mapped read length as one of the authentication criteria, this metric should be considered when assessing samples, particularly when replicates of the same parchment map best to multiple species. Given both material age and the chemical environment skins are exposed to during the parchment-making process, it is expected that DNA from the animal skins would be degraded. If the expected degradation is not reflected in the average read length, the sequenced DNA may be from another source that was not authentic to the parchment (*e*.*g*., if the read lengths of mapped reads are close to the maximum read length of the sequencing conformation). While there is no significant correlation between mean read length and sampling method (rubbing vs. brushing, p = 0.1199; brushing vs. cutting, p = 0.2377; cutting vs. rubbing, p = 0.6994), there are differences in mean read length between parchment documents (p<0.0001 in One-way ANOVA). The Modern parchment, with the largest mean fragment size, is significantly different from all other parchments. Parchment 1840 has the next largest mean fragment size and is significantly different from all other parchments. Parchments 15–311, 1738, 1763, 1785, and 1812 have no significant differences among each other but are significantly different than all other parchments. Lastly, 1894 has the shortest mean fragment size and is significantly different from all other parchments.

In order to assess the mean read length of known surface contaminants, reads mapping to the human mtGenome reference were analyzed. While six samples had no reads mapping to the human reference, the other 42 samples have a mean read length of 149 ± 18 bp, significantly higher (p<0.0001) than the overall mean length of reads mapping to the parchment source animal (119 ± 21 bp). While it is not clear if this human contamination represents modern or aged touch DNA, the longer read lengths as compared to the DNA endogenous to the parchment may be able to help distinguish between authentic parchment DNA and surface contamination.

## Conclusions

The presented workflow, using an existing DNA extraction method and commercially available library construction and hybridization capture kits, provides a straightforward method to enrich mtGenomes from parchment samples. While the destructive sampling method yielded the highest coverage and minimal contamination, both non-destructive methods were also able to generate full mtGenomes. These data support the role of non-destructive sampling as a method for libraries and archives interested in learning more about the source animal of parchment documents to collect cellular material, but not wishing (or permitted) to perform destructive sampling. Given non-destructive sampling via brushing represents the most straight-forward and time effective approach, some modifications for collecting and processing brushing samples in future studies could improve results. Firstly, longer brushing times could be utilized to maximize the cellular material being collected. Secondly, we noted during DNA isolations that the brush heads were not completely submerged in the extraction/lysis buffer during the overnight incubation. Thus, an increased volume of extraction/lysis buffer should be used to accommodate the size of the brush within the extraction tube. Lastly, for those parchments known to have been handled significantly, the sampling area can first be “cleaned” slightly via eraser to remove the surface contamination, then sampled via brushing for downstream processing. This should reduce the amount of contaminant DNA isolated, subsequently prepared into libraries and sequenced, increasing the representation of reads authentic to the parchment source animal. These techniques promise to unlock the important data stored in parchment manuscripts that has previously been inaccessible due to the lack of a reliable nondestructive sampling technique, an advancement that will be of great value to a number of fields in both the sciences and humanities, including zooarchaeology, veterinary science, history, and literary studies. Moreover, the workflow reported in this study could be adapted to other challenging sample types, including samples expected to contain degraded DNA (*e*.*g*., aged museum specimens) and chemically processed material (*e*.*g*., leather), by using baits appropriate to the target animal.

## Supporting information

S1 Table Mitochondrial genome (mtGenome) references sourced from GenBank and used for data analysis. * denotes reference genome was used for hybridization capture bait design.
(PDF)

S2 Table Authentication criteria by sample. Each sample was independently evaluated to determine whether the results accurately represented the parchment source. Black text represents criteria passing thresholds, while red text represents metrics that do not pass authentication thresholds defined in the materials and methods. If a sample did not pass a threshold, it did not get evaluated for the subsequent thresholds, noted with a dash (-).
(PDF)

S3 Table Pairwise comparison of mitochondrial genome (mtGenome) references and consensus mtGenome sequences for all samples passing authentication criteria. The lower left corner shows percent identity, while the upper right corner displays the number of nucleotide gaps between the two mtGenomes compared. References are named with the GenBank Accession number, followed by the scientific name, then the common name. Samples from the present study are named with the Parchment ID, collection method (C = cutting, R = rubbing, B = brushing) and replicate number.
(PDF)

S1 Appendix Consensus mitochondrial genome (mtGenome) sequences of authenticated samples. (FA)
