# Supplementary material for: The development of non-destructive sampling methods of parchment skins for genetic species identification
Source: PLoS One. 2024 Mar 20;19(3):e0299524. doi: 10.1371/journal.pone.0299524 (PMC10954192; doi:10.1371/journal.pone.0299524)
Supplement: S1 Table — * denotes reference genome was used for hybridization capture bait design. (PDF) [file pone.0299524.s001.pdf]

GenBank accession	Scientific name	Common name
MT483645.1	*Alces alces*	moose
HQ223450.1	*Bison bonasus*	European bison
KR350472.1	*Bison priscus*	steppe bison
*NC_006853.1	*Bos taurus*	cow
NC_002008.4	*Canis lupus familiaris*	dog
*NC_005044.2	*Capra hircus*	goat
MT430939.1	*Cervus canadensis nannodes*	tule elk
NC_007704.2	*Cervus elaphus*	red deer
NC_001640.1	*Equus caballus*	horse
NC_001700.1	*Felis catus*	cat
FJ207523.1	*Hemitragus jayakari*	Arabian tahr
NC_012920.1	*Homo sapiens*	human
NC_015247.1	*Odocoileus virginianus*	white tailed deer
*NC_001941.1	*Ovis aries*	sheep
NC_015889.1	*Ovis canadensis*	bighorn sheep
NC_000845.1	*Sus scrofa*	domestic pig
