# Supplementary material for: The development of non-destructive sampling methods of parchment skins for genetic species identification
Source: PLoS One. 2024 Mar 20;19(3):e0299524. doi: 10.1371/journal.pone.0299524 (PMC10954192; doi:10.1371/journal.pone.0299524)
Supplement: S2 Table — Each sample was independently evaluated to determine whether the results accurately represented the parchment source. Black text represents criteria passing thresholds, while red text represents metrics that do not pass authentication thresholds defined in the materials and methods. If a sample did not pass a threshold, it did not get evaluated for the subsequent thresholds, noted with a dash (-). (PDF) [file pone.0299524.s002.pdf]

Authentication criteria		1)	2)	3)	4)
**Sample name**	**Sample type**	**Mean mapping coverage (X)**	**Reference covered (%)**	**GenBank top match**	**Phylogenetic tree placement**
15-311-B1	Brushing	1.9	-	-	-
15-311-B2	Brushing	1.8	-	-	-
15-311-C1	Cutting	50.1	100	*Bos taurus*	*Bos taurus*
15-311-C2	Cutting	80.3	100	*Bos taurus*	*Bos taurus*
15-311-R1	Rubbing	4.4	-	-	-
15-311-R2	Rubbing	11.9	99.3	*Bos taurus*	*Bos taurus*
1738-B1	Brushing	49.7	100	*Ovis aries*	*Ovis aries*
1738-B2	Brushing	69.9	100	*Ovis aries*	*Ovis aries*
1738-C1	Cutting	162.1	100	*Ovis aries*	*Ovis aries*
1738-C2	Cutting	207.5	100	*Ovis aries*	*Ovis aries*
1738-R1	Rubbing	98	100	*Ovis aries*	*Ovis aries*
1738-R2	Rubbing	82	100	*Ovis aries*	*Ovis aries*
1763-B1	Brushing	3.3	-	-	-
1763-B2	Brushing	5.9	-	-	-
1763-C1	Cutting	116	100	*Ovis aries*	*Ovis aries*
1763-C2	Cutting	109.1	100	*Ovis aries*	*Ovis aries*
1763-R1	Rubbing	9.1	-	-	-
1763-R2	Rubbing	8.2	-	-	-
1785-B1	Brushing	34.9	99.5	*Ovis aries*	*Ovis aries*
1785-B2	Brushing	51.4	100	*Ovis aries*	*Ovis aries*
1785-C1	Cutting	266.6	100	*Ovis aries*	*Ovis aries*
1785-C2	Cutting	281.2	100	*Ovis aries*	*Ovis aries*
1785-R1	Rubbing	21.2	99.4	*Ovis aries*	*Ovis aries*
1785-R2	Rubbing	50.3	100	*Ovis aries*	*Ovis aries*
1812-B1	Brushing	2.6	-	-	-
1812-B2	Brushing	3	-	-	-
1812-C1	Cutting	25	99.5	*Ovis aries*	*Ovis aries*
1812-C2	Cutting	44.8	100	*Ovis aries*	*Ovis aries*
1812-R1	Rubbing	5.9	-	-	-
1812-R2	Rubbing	4.6	-	-	-
1840-B1	Brushing	35.2	100	*Ovis aries*	*Ovis aries*
1840-B2	Brushing	46.7	100	*Ovis aries*	*Ovis aries*
1840-C1	Cutting	346.8	100	*Ovis aries*	*Ovis aries*
1840-C2	Cutting	354.3	100	*Ovis aries*	*Ovis aries*
1840-R1	Rubbing	63.4	100	*Ovis aries*	*Ovis aries*
1840-R2	Rubbing	151.2	100	*Ovis aries*	*Ovis aries*
1894-B1	Brushing	3.3	-	-	-
1894-B2	Brushing	16.2	99.1	*Ovis aries*	*Ovis aries*
1894-C1	Cutting	175.4	99.7	*Ovis aries*	*Ovis aries*
1894-C2	Cutting	141.9	100	*Ovis aries*	*Ovis aries*
1894-R1	Rubbing	7.2	-	-	-
1894-R2	Rubbing	7.5	-	-	-
Modern-B1	Brushing	231.2	100	*Bos taurus*	*Bos taurus*
Modern-B2	Brushing	225.9	100	*Bos taurus*	*Bos taurus*
Modern-C1	Cutting	268.7	100	*Bos taurus*	*Bos taurus*
Modern-C2	Cutting	253.4	100	*Bos taurus*	*Bos taurus*
Modern-R1	Rubbing	244.3	100	*Bos taurus*	*Bos taurus*
Modern-R2	Rubbing	315	100	*Bos taurus*	*Bos taurus*
